# Associations of Suicidality Trends With Cannabis Use as a Function of Sex and Depression Status

**DOI:** 10.1001/jamanetworkopen.2021.13025

**Published:** 2021-06-22

**Authors:** Beth Han, Wilson M. Compton, Emily B. Einstein, Nora D. Volkow

**Affiliations:** 1 National Institute on Drug Abuse, National Institutes of Health, Bethesda, Maryland

## Abstract

**Importance:**

During the past decade, cannabis use among US adults has increased markedly, with a parallel increase in suicidality (ideation, plan, attempt, and death). However, associations between cannabis use and suicidality among young adults are poorly understood.

**Objective:**

To determine whether cannabis use and cannabis use disorder (CUD) are associated with a higher prevalence of suicidality among young adults with or without depression and to assess whether these associations vary by sex.

**Design, Setting, and Participants:**

This survey study examined data from 281 650 adults aged 18 to 34 years who participated in the National Surveys on Drug Use and Health. Data were collected from January 1, 2008, to December 31, 2019.

**Exposures:**

Prevalence of past-year daily or near-daily cannabis use (≥300 days in the past year), CUD, and major depressive episode (MDE). Past-year CUD and MDE were based on *DSM-IV* diagnostic criteria.

**Main Outcomes and Measures:**

Past-year suicidal ideation, plan, and attempt.

**Results:**

Among the 281 650 adults aged 18 to 34 (men, 49.9% [95% CI, 49.6%-50.2%]; women, 50.1% [95% CI, 49.8%-50.4%]) included in the analysis, past-year suicidal ideation and plan along with daily cannabis use increased among all examined sociodemographic subgroups (except daily cannabis use among current high-school students), and past-year suicide attempt increased among most subgroups. National trends in adjusted prevalence of past-year suicidal ideation, plan, and attempt varied by daily and nondaily cannabis use and CUD among adults with or without MDE. After controlling for MDE, CUD, cannabis use status, and potential confounding factors, the adjusted prevalence of suicidal ideation, plan, and attempt increased 1.4 to 1.6 times from the 2008-2009 to 2018-2019 periods (adjusted risk ratio [ARR] for suicidal ideation, 1.4 [95% CI, 1.3-1.5]; ARR for suicide plan, 1.6 [95% CI, 1.5-1.9]; ARR for suicide attempt, 1.4 [95% CI, 1.2-1.7]), with 2008 to 2009 as the reference period. Past-year CUD, daily cannabis use, and nondaily cannabis use were associated with a higher prevalence of past-year suicidal ideation, plan, and attempt in both sexes (eg, among individuals without MDE, prevalence of suicidal ideation for those with vs without CUD was 13.9% vs 3.5% among women and 9.9% vs 3.0% among men; *P* < .001), but significantly more so in women than men (eg, suicide plan among those with CUD and MDE was 52% higher for women [23.7%] than men [15.6%]; *P* < .001).

**Conclusions and Relevance:**

From 2008 to 2019, suicidal ideation, plan, and attempt increased 40% to 60% over increases ascribed to cannabis use and MDE. Future research is needed to examine this increase in suicidality and to determine whether it is due to cannabis use or overlapping risk factors.

## Key Points

**Question:**

Are there associations between cannabis use and suicidality trends in young adults, and do they vary as a function of sex and depression?

**Findings:**

This survey study examined 281 650 adult participants in the 2008-2019 National Surveys of Drug Use and Health data and found associations of past-year cannabis use disorder, daily cannabis use, and nondaily cannabis use with higher prevalence of past-year suicidal ideation, plan, and attempt in both sexes, but significantly more in women.

**Meaning:**

In this study, cannabis use was associated with higher prevalence of suicidal ideation, plan, and attempt among US young adults with or without depression, and the risks were greater for women than men.

## Teaser

This survey study assesses whether cannabis use and cannabis use disorder are associated with a higher prevalence of suicidality among young adults with or without depression and whether these associations vary by sex.

## Introduction

By April 2021, 15 US states and Washington, DC, had legalized nonmedical use of cannabis by adults, and 36 states and Washington, DC, had legalized medical use of cannabis. From 2008 to 2019, the number of adults with past-year cannabis use doubled from 22.6 million to 45.0 million. In parallel, the number of adults with cannabis use disorder (CUD) increased from 3.4 million to 4.1 million, and adults with daily or near-daily cannabis use (hereafter daily cannabis use) nearly tripled from 3.6 million to 9.8 million.^1,2^ During the same time frame, the number of adults with a past-year major depressive episode (MDE) increased from 14.5 million to 19.4 million, the number of adults with serious thoughts of suicide (hereafter referred to as suicidal ideation) increased from 8.3 million to 12.0 million,^1,2^ and the number of adults who died by suicide increased from 35 045 to 45 861.^3^

To inform suicide prevention efforts, it is critical to understand the factors that contribute to these increases. Studies have shown that depression is one of the strongest risk factors for suicidal ideation,^4,5,6,7,8,9^ plan,^7,8,10^ and attempt^10,11,12^ and death by suicide.^13,14,15,16^ Depression is associated with CUD^17^ and medical and nonmedical cannabis use.^18,19^ Cannabis use has also been associated with suicidal ideation and attempt^20,21^; in particular, frequent use is associated with suicidal ideation^21,22^ and attempt,^21,23^ and CUD is associated with self-harm^24^ and death by suicide.^25,26^ However, no studies have examined whether and how changes in depression, daily cannabis use, and CUD influence suicidality trends.

Furthermore, although sex differences in depression^27,28,29^ and suicidality^10,30^ are well documented, sex differences in their interactions with cannabis use are less clear. Whereas one population study^31^ reported a stronger association between adolescent cannabis use and adult depression in female vs male participants, another study^32^ found a stronger association between CUD and depressive symptoms in male participants aged 19 to 20 years and in female participants older than 25 years. However, no studies have examined sex differences in the interactions among cannabis use, CUD, and suicidal ideation, plan, and attempt, with or without depression.

To address these knowledge gaps, we used nationally representative data collected from January 1, 2008, to December 31, 2019, and examined adults aged 18 to 34 years, the age range when most substance use disorders and mood disorders emerge.^33^ This study sought to determine whether cannabis use and CUD are associated with increased suicidality risks among young adults with or without depression and to assess whether these associations vary as a function of sex.

## Methods

### Survey Methods and Study Population

The 2008-2019 National Surveys on Drug Use and Health (NSDUH) were face-to-face household interview surveys conducted by the Substance Abuse and Mental Health Services Administration. The annual NSDUH used a stratified, multistage area probability sample that was designed to be representative of the nation and each state. The NSDUH data collection protocol was approved by the institutional review board at RTI International. Data were collected by interviewers in personal visits to households and noninstitutional group quarters. Each participant provided verbal informed consent.^34^

The NSDUH collected nationally representative data among US civilian, noninstitutionalized adult populations on MDE, CUD, daily cannabis use and past-year suicidal ideation, plan, and attempt.^1,34^ Audio computer-assisted, self-administered interviewing was used, providing respondents with a private, confidential way to record answers. The annual mean weighted response rate of the 2008-2019 NSDUH was 58.2%, according to the American Association for Public Opinion Research (AAPOR) reporting guideline for in-person household surveys.^35^

### Measures of Main Outcomes and Participant Characteristics

Among adult respondents, the 2008-2019 NSDUH asked about suicidal ideation, plan, and attempt in the past year.^10,12,36,37^ The NSDUH asked all respondents about lifetime and past-year use of tobacco, alcohol, cannabis, and cocaine and the number of days of use in the past year. Past-year cannabis use status was categorized as past-year daily or near daily cannabis use (hereafter daily cannabis use, ≥300 days), nondaily cannabis use, and no cannabis use.

Using *DSM-IV* diagnostic criteria, the NSDUH estimated prevalence of past-year specific alcohol, cannabis, and cocaine use disorders^38^ and MDE.^29^ Nicotine dependence was assessed using the Nicotine Dependence Syndrome Scale.^39^ These measures of substance use and substance use disorders have demonstrated good validity and reliability.^40,41,42^ The NSDUH also queried sociodemographic characteristics (eg, age, sex, race/ethnicity, educational attainment, college/school enrollment, employment status, family income, marital status, and health insurance). Race/ethnicity was the NSDUH respondent’s self-classification of racial/ethnic origin and identification based on the classifications developed by the US Census Bureau.

### Statistical Analysis

First, we estimated and tested trends in past-year suicidal ideation, plan, and attempt and CUD and daily cannabis use among adults aged 18 to 34 years by sociodemographic characteristics (and by MDE, CUD, and cannabis use status for suicidality trends) from 2008 to 2019. Second, to assess correlates of past-year suicidal ideation, plan, and attempt, we applied multivariable logistic regression modeling and tested multicollinearity and potential interaction effects. Third, because of significant interaction effects between sex and/or MDE and other covariates (eg, CUD and cannabis use status) identified in pooled models for suicidality outcomes, we stratified multivariable logistic regression analyses by sex, MDE, and CUD and cannabis use status to examine trends in model-adjusted prevalence^43^ of suicidal ideation, plan, and attempt, adjusting for sociodemographic characteristics, nicotine dependence, alcohol use disorder, and cocaine use disorder. Fourth, sex differences were estimated and tested in model-adjusted prevalence (adjusted risk differences^43^) of suicidal ideation, plan, and attempt by MDE, CUD, and cannabis use status, controlling for survey year and other covariates above. All analyses used SUDAAN software, version 11.0.3,^44^ to account for the complex sample design and sample weights of the NSDUH. For all analyses, *P <* .05 (2-tailed) was considered statistically significant.

## Results

### Trends in Prevalence of Past-Year Suicidality

Among the 281 650 sampled adults aged 18 to 34 years from the 2008-2019 NSDUH, 49.9% (95% CI, 49.6%-50.2%) were male and 50.1% (95% CI, 49.8%-50.4%) were female. Among US adults aged 18 to 34 from 2008 to 2019, prevalence of past-year suicidal ideation and suicide plan increased for every examined sociodemographic subgroup, and prevalence of past-year suicide attempt increased for most examined sociodemographic subgroups (eTables 1-3 in the Supplement). Within subgroups in 2018 to 2019, a relatively higher prevalence (SE) of past-year suicidal ideation, plan, and attempt was observed among adults aged 18 to 23 (12.4 [0.3], 3.9 [0.2], and 2.0 [0.1], respectively), women (9.9 [0.3], 3.2 [0.1], and 1.5 [0.1], respectively), individuals with annual family income less than $20 000 (11.3 [0.4], 3.8 [0.2], and 1.8 [0.1], respectively), and adults with MDE and daily cannabis use (52.6 [3.1], 22.4 [2.2], and 9.6 [1.5], respectively) or with CUD (50.8 [3.1], 20.5 [2.7], and 10.8 [1.7], respectively) (Table 1).

**Table 1.** Prevalence of Past-Year Serious Suicidality, Suicide Plan, Suicide Attempt, CUD, and Daily or Near-Daily CU Among US Adults Aged 18 to 34

Characteristic	Prevalence, weighted % (SE)^a^				
	Suicidal ideation (n = 44 807)	Suicide plan (n = 44 799)	Suicide attempt (n = 44 798)	Daily/near daily CU (n = 45 258)	CUD (n = 45 258)
Overall	8.7 (0.2)	2.7 (0.1)	1.2 (0.1)	7.0 (0.2)	4.2 (0.2)
Age, y					
18-23^b^	12.4 (0.3)	3.9 (0.2)	2.0 (0.1)	7.5 (0.2)	6.4 (0.3)
24-29	8.2 (0.3)^c^	2.7 (0.2)^c^	1.0 (0.1)^c^	7.7 (0.3)	3.8 (0.3)^c^
30-34	5.3 (0.3)^c^	1.4 (0.2)^c^	0.6 (0.1)^c^	5.6 (0.4)^c^	2.0 (0.2)^c^
Sex					
Men^b^	7.6 (0.2)	2.2 (0.1)	1.0 (0.1)	8.9 (0.3)	5.4 (0.2)
Women	9.9 (0.3)^c^	3.2 (0.1)^c^	1.5 (0.1)^c^	5.0 (0.2)^c^	2.9 (0.2)^c^
Race/ethnicity					
Non-Hispanic White^b^	9.7 (0.3)	2.9 (0.1)	1.1 (0.1)	7.6 (0.3)	4.1 (0.2)
Non-Hispanic Black	7.0 (0.4)^c^	2.5 (0.2)	1.5 (0.2)^c^	8.7 (0.4)^c^	4.9 (0.4)
Hispanic	7.7 (0.4)^c^	2.4 (0.2)^c^	1.2 (0.2)	5.3 (0.4)^c^	3.9 (0.3)
Non-Hispanic other	8.0 (0.5)^c^	2.7 (0.2)	1.4 (0.2)	5.1 (0.4)^c^	3.8 (0.4)
Family income, $					
<20 000^b^	11.3 (0.4)	3.8 (0.2)	1.8 (0.1)	7.9 (0.4)	5.3 (0.4)
20 000-49 999	9.2 (0.3)^c^	3.0 (0.2)^c^	1.3 (0.1)^c^	7.9 (0.3)	3.9 (0.2)^c^
50 000-74 999	7.7 (0.4)^c^	2.3 (0.2)^c^	0.9 (0.1)^c^	7.5 (0.4)	3.9 (0.3)^c^
≥75 000	7.4 (0.3)^c^	2.0 (0.1)^c^	0.9 (0.1)^c^	5.4 (0.3)^c^	3.8 (0.2)^c^
Employment status					
Full-time employed	7.4 (0.2)^c^	2.1 (0.1)^c^	0.9 (0.1)^c^	7.2 (0.2)^c^	3.5 (0.2)^c^
Part-time employed	11.2 (0.5)	3.3 (0.2)	1.2 (0.1)^c^	6.7 (0.4)^c^	5.2 (0.3)^c^
Unemployed^b^	11.8 (0.8)	4.3 (0.4)	2.9 (0.4)	11.3 (0.7)	8.0 (0.8)
Other	9.2 (0.4)^c^	3.4 (0.3)	1.6 (0.2)^c^	5.0 (0.3)^c^	3.5 (0.2)^c^
College/school enrollment					
Full-time college student	10.4 (0.4)^c^	2.8 (0.2)^c^	1.2 (0.1)^c^	4.8 (0.3)	5.2 (0.4)^c^
Part-time college student	11.5 (0.8)^c^	3.8 (0.4)^c^	1.6 (0.3)^c^	6.2 (0.6)^c^	4.0 (0.3)^c^
College graduate, no enrollment^b^	5.7 (0.4)	1.2 (0.2)	0.3 (0.1)	4.3 (0.3)	2.8 (0.3)
Some college education, no enrollment now	10.1 (0.4)^c^	3.2 (0.3)^c^	1.3 (0.2)^c^	9.6 (0.5)^c^	4.6 (0.3)^c^
High school graduate, no enrollment now	8.9 (0.4)^c^	3.4 (0.3)^c^	1.6 (0.1)^c^	9.1 (0.4)^c^	4.3 (0.3)^c^
Current high school students	10.2 (1.1)^c^	4.5 (0.8)^c^	2.5 (0.6)^c^	4.7 (0.6)	4.8 (0.6)^c^
Less than high school education, no enrollment now	7.8 (0.5)^c^	2.8 (0.3)^c^	2.0 (0.3)^c^	8.2 (0.6)^c^	4.4 (0.5)^c^
Past-year daily CU/MDE status					
Daily CU/MDE	52.6 (3.1)^c^	22.4 (2.2)^c^	9.6 (1.5)^c^	NA	NA
Daily CU/no MDE	9.2 (0.7)^c^	2.2 (0.3)^c^	1.2 (0.2)^c^	NA	NA
Nondaily CU/MDE	43.5 (1.5)^c^	15.7 (1.1)^c^	7.1 (0.7)^c^	NA	NA
Nondaily CU/no MDE	6.8 (0.3)^c^	1.7 (0.2)^c^	0.9 (0.1)^c^	NA	NA
No past-year CU/MDE	35.0 (1.0)^c^	12.7 (0.9)^c^	4.5 (0.4)^c^	NA	NA
No past-year CU/no MDE	3.3 (0.2)	0.7 (0.1)	0.4 (0.1)	NA	NA
Past-year CUD/MDE status					
CUD/MDE	50.8 (3.1)^c^	20.5 (2.7)^c^	10.8 (1.7)^c^	NA	NA
CUD/no MDE	14.0 (1.0)^c^	4.0 (0.6)^c^	2.1 (0.4)^c^	NA	NA
No CUD/MDE	38.9 (0.9)^c^	14.2 (0.6)^c^	5.5 (0.4)^c^	NA	NA
No CUD/no MDE^b^	4.1 (0.1)	0.9 (0.1)	0.5 (0.1)	NA	NA
Past-year CUD					
Yes	NA	NA	NA	45.5 (1.5)^c^	NA
No^b^	NA	NA	NA	5.3 (0.1)	NA
Past-year daily CU					
Yes	NA	NA	NA	NA	27.0 (1.2)^c^
No, but CU in the past year^b^	NA	NA	NA	NA	9.5 (0.4)
Past-year MDE					
Yes	NA	NA	NA	11.3 (0.6)^c^	9.6 (0.5)^c^
No^b^	NA	NA	NA	6.3 (0.2)	3.4 (0.1)
Past-year suicidality					
Yes	NA	NA	NA	14.2 (0.8)^c^	11.7 (0.7)^c^
No^b^	NA	NA	NA	6.3 (0.2)	3.4 (0.2)
Past-year suicide plan					
Yes	NA	NA	NA	16.2 (1.3)^c^	13.5 (1.3)^c^
No^b^	NA	NA	NA	6.7 (0.2)	3.9 (0.2)
Past-year suicide attempt					
Yes	NA	NA	NA	16.6 (1.9)^c^	15.6 (1.8)^c^
No^b^	NA	NA	NA	6.8 (0.2)	4.0 (0.2)

Abbreviations: CU, cannabis use; CUD, cannabis use disorder; MDE, major depressive episode; NA, not applicable; SE, standard error.

^a^ From the 2018-2019 National Surveys on Drug Use and Health.

^b^ Indicates reference group.

^c^ *P* < .05 compared with the estimate of the reference group.

### Trends in Prevalence of Past-Year Daily Cannabis Use and CUD

Among US adults aged 18 to 34 years, prevalence of past-year daily cannabis use increased for every examined sociodemographic group (except no change among those who were current high school students) from 2008 to 2019 (eTable 4 in the Supplement). Prevalence of daily cannabis use also increased among adults with or without MDE. Within subgroups in 2018 to 2019, a relatively higher prevalence (SE) of past-year daily cannabis use was found among adults aged 18 to 29 years (age 18-23 years, 7.5 [0.2]; age 24-29 years,7.7 [0.3]), men (8.9 [0.3]), non-Hispanic Black individuals (8.7 [0.4]), unemployed adults (11.3 [0.7]), individuals with CUD (45.5 [1.5]), individuals with MDE (11.3 [0.6]), individuals with suicidal ideation (14.2 [0.8]), individuals with suicide plan (16.2 [1.3]), and individuals with suicide attempt (16.6 [1.9]) (Table 1).

By contrast, the prevalence of past-year CUD remained stable from 2008 to 2019 (eTable 5 in the Supplement). However, within subgroups, prevalence (SE) increased among individuals aged 24 to 29 (from 3.0 [0.2] to 3.8 [0.3]), individuals with annual family income from $50 000 to $74 999 (from 2.9 [0.3] to 3.9 [0.3]), and individuals with full-time employment (from 3.0 [0.1] to 3.5 [0.2]). The prevalence of past-year CUD decreased among adults with daily cannabis use (from 36.6 [1.39] to 27.0 [1.16]) and among adults without MDE (from 3.6 [0.13] to 3.4 [0.14]).

### Trends in Adjusted Past-Year Suicidality by Sex, MDE, CUD, and Cannabis Use

eTable 6 in the Supplement shows the results of the final pooled multivariable logistic regression models for suicidal ideation, plan, and attempt. Consistently, after controlling for MDE, CUD, cannabis use status, and potential confounding factors, the adjusted prevalence of suicidal ideation, plan, and attempt increased 1.4 to 1.6 times from the 2008-2009 to 2018-2019 periods (adjusted risk ratio [ARR] for suicidal ideation, 1.4 [95% CI, 1.3-1.5]; ARR for suicide plan, 1.6 [95% CI, 1.5-1.9]; and ARR for suicide attempt, 1.4 [95% CI, 1.2-1.7]).

In these pooled models, however, we identified several interaction effects between sex and/or MDE and other covariates (eg, 3-way interaction effect of sex, MDE, and CUD on suicidal ideation [*P* < .001], plan [*P* < .001], and attempt [*P* = .05]; 3-way interaction effect of sex, MDE, and cannabis use status on suicidal ideation [*P* = .003], plan [*P* = .001], and attempt [*P* = .01]). To better understand how these trends in suicidal outcomes varied by depression, cannabis use, and sex, we stratified multivariable logistic regression analyses by sex, MDE, and CUD and cannabis use status.

Table 2 presents the trends in adjusted prevalence of past-year suicidal ideation by sex, MDE, and CUD and cannabis use status. From 2008 to 2019, the adjusted prevalence of suicidal ideation increased among men with MDE but without CUD, among men without MDE, and among women with or without MDE. In particular, prevalence (95% CI) increased among women with MDE and CUD, from 40.7% (32.2%-49.7%) to 56.6% (49.0%-63.9%); with MDE and without CUD, from 28.1% (25.4%-31.1%) to 38.1% (36.0%-40.4%); with MDE and daily cannabis use, from 40.6% (29.0%-53.3%) to 55.0% (48.1%-61.7%); with MDE and nondaily cannabis use, from 34.9% (29.8%-40.4%) to 46.7% (42.8%-50.5%); with MDE and without cannabis use, from 25.0% (22.2%-28.1%) to 34.1% (31/5%-36.9%); without MDE and with CUD, from 10.7% (7.2%-15.6%) to 18.4% (14.0%-23.9%); without MDE and CUD, from 2.9% (2.6%-3.2%) to 4.4% (4.0%-4.7%); without MDE and with daily cannabis use, from 7.4% (4.8%-11.8%) to 13.2% (10.2%-16.7%); without MDE and with nondaily cannabis use, from 5.2% (4.4%-6.1%) to 9.0% (7.9%-10.3%); and without MDE and cannabis use (from 2.4% (2.1%-2.7%) to 3.3% (2.9%-3.7%). 

**Table 2.** Adjusted Prevalence of Past-Year Suicidal Ideation Among US Adults Aged 18 to 34 Years^a^

Study period	Prevalence, weighted % (95% CI)									
	With MDE					Without MDE				
	CUD^b^	No CUD^b^	Daily/near-daily CU^c^	Nondaily CU^c^	No CU^c^	CUD^b^	No CUD^b^	Daily/near-daily CU^c^	Nondaily CU^c^	No CU^c^
**For men**										
β coefficient	0.097	0.053	0.091	0.056	0.048	0.138	0.117	0.097	0.140	0.112
*P* value	.07	.03	.19	.07	.11	<.001	<.001	.01	<.001	<.001
2008-2009^d^	44.5 (33.8-55.8)	36.1 (31.4-41.2)	49.4 (35.6-63.3)	40.7 (33.5-48.4)	33.7 (27.9-40.1)	8.3 (6.5-10.6)	2.4 (2.1-2.8)	6.6 (5.0-8.6)	3.3 (2.7-4.1)	2.1 (1.7-2.5)
2010-2011	40.0 (30.8-49.9)	34.7 (30.0-39.7)	42.0 (27.3-58.3)	36.3 (29.5-43.6)	32.4 (27.4-37.8)	7.9 (6.2-10.0)	2.6 (2.3-2.9)	5.7 (4.52-7.2)	4.2 (3.3-5.2)	2.1 (1.8-2.5)
2012-2013	42.3 (31.4-54.0)	34.3 (30.2-38.6)	46.7 (34.4-59.4)	37.1 (30.9-43.8)	31.1 (26.0-36.8)	8.0 (5.9-10.7)	3.0 (2.6-3.4)^e^	7.4 (5.8-9.4)	4.4 (3.4-5.7)	2.5 (2.1-2.9
2014-2015	42.3 (32.5-52.7)	36.8 (33.6-40.2)	46.0 (35.2-57.1)	44.8 (38.6-51.2)	30.8 (27.5-34.2)	10.4 (7.9-13.6)	3.3 (2.9-3.7)^e^	6.1 (4.6-8.0)	5.5 (4.6-6.5)^e^	2.8 (2.4-3.2)^e^
2016-2017	53.2 (43.0-63.1)	38.3 (35.0-41.8)	42.6 (34.0-51.7)	44.3 (38.9-49.9)	36.6 (33.0-40.4)	12.5 (10.0-15.4)^e^	3.9 (3.4-4.4)^e^	10.1 (8.2-12.4)^e^	6.0 (5.0-7.2)^e^	3.2 (2.7-3.7)^e^
2018-2019	50.9 (43.9-57.9)	40.7 (38.5-43.0)	55.9 (48.2-63.4)	43.5 (39.3-47.8)	37.9 (35.1-40.8)	13.8 (11.3-16.7)^e^	4.0 (3.6-4.4)^e^	8.6 (6.9-10.8)	6.5 (5.7-7.4)^e^	3.4 (2.9-3.9)
**Women**										
β coefficient	0.135	0.109	0.109	0.122	0.102	0.105	0.083	0.140	0.100	0.068
*P* value	.01	<.001	.03	<.001	<.001	.04	<.001	.01	<.001	<.001
2008-2009^d^	40.7 (32.2-49.7)	28.1 (25.4-31.1)	40.6 (29.0-53.3)	34.9 (29.8-40.4)	25.0 (22.2-28.1)	10.7 (7.2-15.6)	2.9 (2. 6-3.2)	7.4 (4.8-11.8)	5.2 (4.4-6.1)	2.4 (2.1-2.7)
2010-2011	48.9 (38.6-59.2)	27.9 (25.7-30.2)	48.2 (35.9-60.6)	33.6 (29.1-38.5)	25.9 (23.3-28.8)	11.5 (8.7-15.2)	2.9 (2.6-3.2)	7.0 (5.3-9.3)	5.5 (4.5-6.7)	2.3 (2.1-2.7)
2012-2013	47.8 (37.0-58.8)	27.6 (25.1-30.3)	42.7 (33.7-52.1)	32.5 (28.4-36.9)	25.9 (23.0-28.9)	16.3 (12.1-21.6)^e^	3.1 (2.9-3.5)	9.2 (7.0-12.1)	6.8 (5.5-8.3)^e^	2.5 (2.2-2.8)
2014-2015	52.5 (42.8-62.1)	32.3 (29.8-34.9)^e^	46.3 (37.0-55.8)	38.9 (34.0-44.0)	29.6 (26.9-32.4)^e^	11.9 (9.4-15.0)	3.3 (3.0-3.6)	9.7 (7.4-12.6)	6.6 (5.6-7.7)^e^	2.6 (2.3-2.9)
2016-2017	57.6 (49.6-65.2)^e^	35.3 (33.0-37.7)^e^	47.2 (39.7-54.8)	42.2 (38.9-45.6)^e^	33.0 (30.4-35.7)^e^	13.5 (10.4-17.4)	3.4 (3.1-3.8)^e^	10.5 (8.4-13.1)	5.9 (4.9-7.1)	2.9 (2.5-3.2)
2018-2019	56.6 (49.0-63.9)^e^	38.1 (36.0-40.4)^e^	55.0 (48.1-61.7)^e^	46.7 (42.8-50.5)^e^	34.1 (31.5-36.9)^e^	18.4 (14.0-23.9)^e^	4.4 (4.0-4.7)^e^	13.2 (10.2-16.7)^e^	9.0 (7.9-10.3)^e^	3.3 (2.9-3.7)^e^

Abbreviations: CU, cannabis use; CUD, cannabis use disorder; MDE, major depressive episode.

^a^ From the 2008-2019 National Surveys on Drug Use and Health. Includes 279 886 participants.

^b^ Controlled for age, race/ethnicity, family income, employment, college enrollment, marital status, health insurance, nicotine dependence, alcohol use disorder, cocaine use disorder, and daily or near-daily CU.

^c^ Controlled for age, race/ethnicity, family income, employment, college enrollment, marital status, health insurance, nicotine dependence, alcohol use disorder, cocaine use disorder, and CUD (excluded in models for those without CU).

^d^ Indicates reference group.

^e^ *P* < .05 compared with the estimate of the reference group.

Table 3 presents the trends in adjusted prevalence of past-year suicide plan among young adults by sex, MDE, and CUD and cannabis use status. From 2008 to 2009 and 2018 to 2019, the adjusted prevalence (95% CI) of suicide plan increased among men with MDE and nondaily cannabis use, from 10.3% (7.2%-14.6%) to 17.0% (13.8%-20.9%); among men without MDE with CUD, from 2.1% (1.3%-3.5%) to 4.8% (3.2%-7.7%); among men without MDE and CUD, from 0.4% (0.3%-0.5%) to 0.9% (0.8%-1.1%); among men without MDE and with nondaily cannabis use, from 0.7% (0.5%-1.0%) to 1.7% (1.3%-2.2%); and among men without MDE and cannabis use, from 0.3% (0.2%-0.4%) to 0.8% (0.6%-1.0%). Among women with MDE, prevalence (95% CI) increased for those without CUD, from 9.3% (8.0%-10.8%) to 14.4% (12.9%-16.1%); with daily cannabis use, from 14.5% (8.4%-24.1%) to 26.8% (20.6%-34.0%); with nondaily cannabis use, from 12.0% (9.6%-14.9%) to 17.5% (14.7%-20.7%); and without cannabis use, from 8.2% (7.0%-9.7%) to 13.1% (11.2%-15.3%). Among women without MDE, prevalence (95% CI) increased for those with CUD, from 2.4% (1.3%-4.5%) to 5.3% (3.5%-8.0%); those without CUD, from 0.5% (0.4%-0.7%) to 1.1% (0.9%-1.3%); those with nondaily cannabis use, from 1.0% (0.7%-1.6%) to 2.3% (1.8%-3.1%); and those without cannabis use, from 0.4% (0.3%-0.6%) to 0.8% (0.6%-0.9%).

**Table 3.** Adjusted Prevalence of Past-Year Suicide Plan Among US Adults Aged 18 to 34 Years

Study period	Adjusted prevalence, weighted % (95% CI)^a^										
	With MDE					Without MDE					
	CUD^b^	No CUD^b^	Daily/near-daily CU^c^	Nondaily CU^c^	No CU^c^	CUD^b^	No CUD^b^	Daily/near-daily CU^c^	Nondaily CU^c^	No CU^c^
**Men**											
β coefficient	0.107	0.061	0.162	0.101	0.022	0.193	0.139	0.118	0.163	0.141
*P* value	.17	.08	.05	.01	.62	<.001	<.001	.05	<.001	<.001
2008-2009^d^	11.9 (6.1-21.9)	13.2 (10.0-17.1)	14.9 (7.1-28.7)	10.3 (7.2-14.6)	14.2 (10.4-19.1)	2.1 (1.3-3.5)	0.4 (0.3-0.5)	1.6 (0.9-2.7)	0.7 (0.5-1.0)	0.3 (0.2-0.4)
2010-2011	13.5 (8.2-21. 5)	10.5 (8.3-13.3)	8.5 (4.8-14.5)	13.0 (9.8-17.0)	9.9 (7.5-13.1)	2.1 (1.4-2.9)	0.6 (0.5-0.7)^e^	1.3 (0.8-2.1)	1.0 (0.7-1.3)	0.5 (0.3-0.6)
2012-2013	15.8 (10.1-23.8)	12.2 (9.8-15.1)	17.1 (11.8-24.1)	14.4 (10.3-19.6)	10.2 (7.5-13.7)	1.9 (1.2-2.9)	0.8 (0.6-1.0)^e^	2.0 (1.2-3.3)	1.2 (0.8-1.7)^e^	0.6 (0.5-0.9)^e^
2014-2015	16.2 (11.0-23.1)	13.1 (10.3-16.5)	17.0 (10.3-26.7)	14.6 (10.3-20.3)	11.7 (8.6-15.8)	2.4 (1.4-3.9)	0.7 (0.6-0.9)^e^	1.1 (0.7-1.9)	1.6 (1.1-2.2)^e^	0.5 (0.4-0.7)^e^
2016-2017	15.1 (11.0-20.4)	14.7 (12.4-17.3)	16.9 (11.6-24.0)	15.5 (11.6-20.5)	13.6 (11.1-16.7)	3.7 (2.4-5.8)	0.8 (0.7-1.0)^e^	2.7 (1.8-4.0)	1.3 (1.0-1.8)^e^	0.6 (0.5-0.9)^e^
2018-2019	19.2 (12.9-27.5)	15.1 (12.9-17.6)	21.9 (16.0-29.2)	17.0 (13.8-20.9)^e^	13.2 (10.6-16.3)	4.8 (3.2-7.2)^e^	0.9 (0.8-1.1)^e^	2.3 (1.6-3.4)	1.7 (1.3-2.2)^e^	0.8 (0.6-1.0)^e^
**Women**										
β coefficient	0.102	0.113	0.152	0.095	0.111	0.156	0.138	0.135	0.152	0.127
*P* value	.06	<.001	.01	.003	<.001	.02	<.001	.14	<.001	<.001
2008-2009^d^	16.6 (11.7-23.1)	9.3 (8.0-10.8)	14.5 (8.4-24.1)	12.0 (9.6-14.9)	8.2 (7.0-9.7)	2.4 (1.3-4.5)	0.5 (0.4-0.7)	1.7 (0.7-4.3)	1.0 (0.7-1.6)	0.4 (0.3-0.6)
2010-2011	21.4 (15.2-29.4)	9.9 (8.3-11.7)	17.6 (11.3-26.2)	12.9 (9.6-16.9)	9.0 (7.3-11.1)	3.3 (2.1-5.3)	0.6 (0.5-0.8)	2.6 (1.6-4.5)	1.0 (0.7-1.5)	0.5 (0.4-0.7)
2012-2013	27.5 (19.3-37.6)^e^	10.0 (8.5-11.7)	19.7 (13.3-28.1)	13.6 (11.0-16.7)	9.4 (7.6-11.6)	4.0 (1.9-8.1)	0.8 (0.6-0.9)	3.3 (1.6-6.7)	1.9 (1.3-2.9)^e^	0.5 (0.4-0.6)
2014-2015	22.6 (16.4-30.3)	11.0 (9.3-12.9)	22.6 (16.1-30.9)	14.2 (11.8-16.9)	9.4 (7.7-11.5)	4.7 (3.1-7.0)	0.8 (0.7-1.0)^e^	2.0 (1.2-3.3)	2.0 (1.4-2.7)^e^	0.6 (0.5-0.8)
2016-2017	22.4 (16.2-30.1)	13.7 (12.1-15.4)^e^	21.5 (16.1-28.2)	16.4 (13.9-19.4)^e^	12.4 (10.6-14.4)^e^	5.1 (3.3-7.7)	1.0 (0.8-1.2)^e^	4.1 (2.7-6.1)	1.6 (1.2-2.2)	0.8 (0.7-1.1)^e^
2018-2019	28.0 (21.5-35.5)^e^	14.4 (12.9-16.1)^e^	26.8 (20.6-34.0)^e^	17.5 (14.7-20.7)^e^	13.1 (11.2-15.3)^e^	5.3 (3.5-8.0)^e^	1.1 (0.9-1.3)^e^	3.8 (2.5-5.7)	2.3 (1.8-3.1)^e^	0.8 (0.6-0.9)^e^

Abbreviations: CU, cannabis use; CUD, cannabis use disorder; MDE, major depressive episode.

^a^ From the 2008-2019 National Surveys on Drug Use and Health. Includes 279 861 participants.

^b^ Controlled for age, race/ethnicity, family income, employment, college enrollment, marital status, health insurance, nicotine dependence, alcohol use disorder, cocaine use disorder, and daily or near-daily CU.

^c^ Controlled for age, race/ethnicity, family income, employment, college enrollment, marital status, health insurance, nicotine dependence, alcohol use disorder, cocaine use disorder, and CUD (excluded in models for those without CU).

^d^ Indicates reference group.

^e^ *P* < .05 compared with the estimate of the reference group.

Table 4 presents the trends in adjusted prevalence of past-year suicide attempt by sex, MDE, CUD, and cannabis use status. From 2008 to 2019, the adjusted prevalence (95% CI) of suicide attempt increased among men without MDE for those without CUD, from 0.3% (0.2%-0.3%) to 0.5% (0.4%-0.7%); those with nondaily cannabis use, from 0.4% (0.3%-0.7%) to 0.8% (0.6%-1.2%) in 2014-2016; and those without cannabis use, from 0.2% (0.1%-0.3%) to 0.5% (0.4%-0.7%). Prevalence (95% CI) increased among women with MDE and CUD, from 10.4% (7.4%-14.6%) to 18.4% (13.7%-24.4%) and among women with neither MDE nor CUD, from 0.3% (0.3%-0.4%) to 0.5% (0.5%-0.7%).

**Table 4.** Adjusted Prevalence of Past-Year Suicide Attempt Among US Adults Aged 18 to 34 Years

Study period	Adjusted prevalence, weighted % (95% CI)^a^									
	With MDE					Without MDE				
	CUD^b^	No CUD^b^	Daily/near-daily CU^c^	Nondaily CU^c^	No CU^c^	CUD^b^	No CUD^b^	Daily/near-daily CU^c^	Nondaily CU^c^	No CU^c^
**Men**										
β coefficient	0.003	−0.004	0.011	0.012	−0.025	0.132	0.178	0.113	0.142	0.214
*P* value	.98	.93	.91	.82	.68	.10	<.001	.18	.004	<.001
2008-2010^d^	10.2 (5.4-18.3)	6.1 (4.6-8.1)	9.2 (4.1-19.2)	8.4 (5.6-12.3)	5.2 (3.4-7.8)	2.0 (1.4-2.8)	0.3 (0.2-0.3)	1.2 (0.8-1.8)	0.4 (0.3-0.7)	0.2 (0.1-0.3)
2011-2013	6.5 (3.6-11.4)	4.7 (3.5-6.3)	7.1 (4.3-11.5)	5.7 (3.9-8.2)	3.9 (2.7-5.5)	1.5 (1.0-2.4)	0.3 (0.3-0.4)	0.6 (0.4-1.1)^e^	0.7 (0.5-0.8)	0.3 (0.2-0.4)
2014-2016	9.3 (5.8-14.5)	6.2 (5.1-7.6)	9.0 (5.9-13.5)	7.5 (5.6-10.0)	5.5 (4.1-7.3)	2.0 (1.2-3.3)	0.4 (0.4-0.6)^e^	1.7 (1.2-2.5)	0.8 (0.6-1.2)^e^	0.4 (0.3-0.5)^e^
2017-2019	9.8 (6.9-13.6)	5.8 (4.9-7.0)	7.9 (5.7-10.7)	8.5 (7.1-10.2)	4.5 (3.3-6.0)	2.4 (1.7-3.4)	0.5 (0.4-0.7)^e^	1.2 (0.8-1.8)	0.7 (0.5-1.0)	0.5 (0.4-0.7)^e^
**Women**										
β coefficient	0.196	0.050	0.052	0.067	0.070	0.081	0.092	0.190	0.078	0.081
*P* value	.004	.07	.53	.08	.06	.31	.01	.08	.15	.06
2008-2010^d^	10.4 (7.4-14.6)	4.7 (4.0-5.4)	12.7 (7.8-19.9)	6.3 (4.8-8.2)	3.7 (3.1-4.6)	2.1 (1.3-3.6)	0.3 (0.3-0.4)	0.8 (0.4-1.8)	0.7 (0.5-1.1)	0.3 (0.2-0.4)
2011-2013	12.0 (8.0-17.6)	5.1 (4.2-6.3)	11.4 (7.4-17.1)	8.1 (6.3-10.4)	3.9 (3.0-5.2)	2.8 (1.5-5.3)	0.5 (0.4-0.7)^e^	1.8 (0.8-3.9)	1.4 (0.9-2.2)^e^	0.3 (0.2-0.5)
2014-2016	14.2 (10.4-19.1)	5.4 (4.5-6.4)	10.5 (7.3-14.8)	8.8 (7.0-11.1)	4.2 (3.4-5.2)	2.7 (1.9-3.9)	0.5 (0.4-0.6)^e^	1.8 (1.1-2.9)	1.2 (0.9-1.6)^e^	0.4 (0.3-0.5)^e^
2017-2019	18.4 (13.7-24.4)^e^	5.8 (4.9-6.9)	12.2 (8.7-16.9)	8.7 (7.1-10.7)	5.0 (3.9-6.4)	3.9 (2.5-6.1)	0.5 (0.5-0.7)^e^	2.4 (1.5-3.9)^e^	1.2 (0.9-1.6)	0.4 (0.3-0.5)^e^

Abbreviations: CU, cannabis use; CUD, cannabis use disorder; MDE, major depressive episode.

^a^ From the 2008-2019 National Surveys on Drug Use and Health. Includes 279 856 participants.

^b^ Controlled for age, race/ethnicity, family income, employment, college enrollment, marital status, health insurance, nicotine dependence, alcohol use disorder, cocaine use disorder, and daily or near-daily CU.

^c^ Controlled for age, race/ethnicity, family income, employment, college enrollment, marital status, health insurance, nicotine dependence, alcohol use disorder, cocaine use disorder, and CUD (excluded in models for those without CU).

^d^ Indicates reference group.

^e^ *P* < .05 compared with the estimate of the reference group.

### Sex Differences in Suicidality by MDE, CUD, and Cannabis Use Status

To investigate whether sex differences in suicidal ideation, plan, and attempt varied by depression and cannabis use, we conducted multivariable logistic regression models stratified by MDE, CUD, and cannabis use status and estimated and tested sex differences in these suicidality outcomes. The Figure, A, shows that the adjusted prevalence of past-year suicidal ideation was higher among women with CUD with MDE (52.2% vs 46.1%) and without MDE (13.9% vs 9.9%); among women with daily (10.1% vs 7.4%) or nondaily cannabis use (6.9% vs 4.8%), but without MDE; and among women with neither MDE nor CUD (3.5% vs 3.0%) compared with their male counterparts. However, among women with MDE without CUD (32.7% vs 36.2%) and without cannabis use (29.5% vs 33.3%), the adjusted prevalence was lower than that among their male counterparts. The Figure, B and C, illustrates that the adjusted prevalence of past-year suicide plan and suicide attempt was higher among women with MDE with CUD (23.7% vs 15.6% and 13.7% vs 9.2%, respectively) or daily cannabis use (21.8% vs 17.4% and 11.7% vs 8.1%, respectively) and among women without MDE with CUD (4.1% vs 2.7% and 3.0% vs 1.5%, respectively) or without CUD (0.8% vs 0.7% and 0.5% vs 0.4%, respectively) and with daily cannabis use (3.2% vs 1.8% and 2.0% vs 1.0%, respectively) or nondaily cannabis use (1.8% vs 1.2% and 1.2% vs 0.6%, respectively) compared with their male counterparts.

**Figure.**
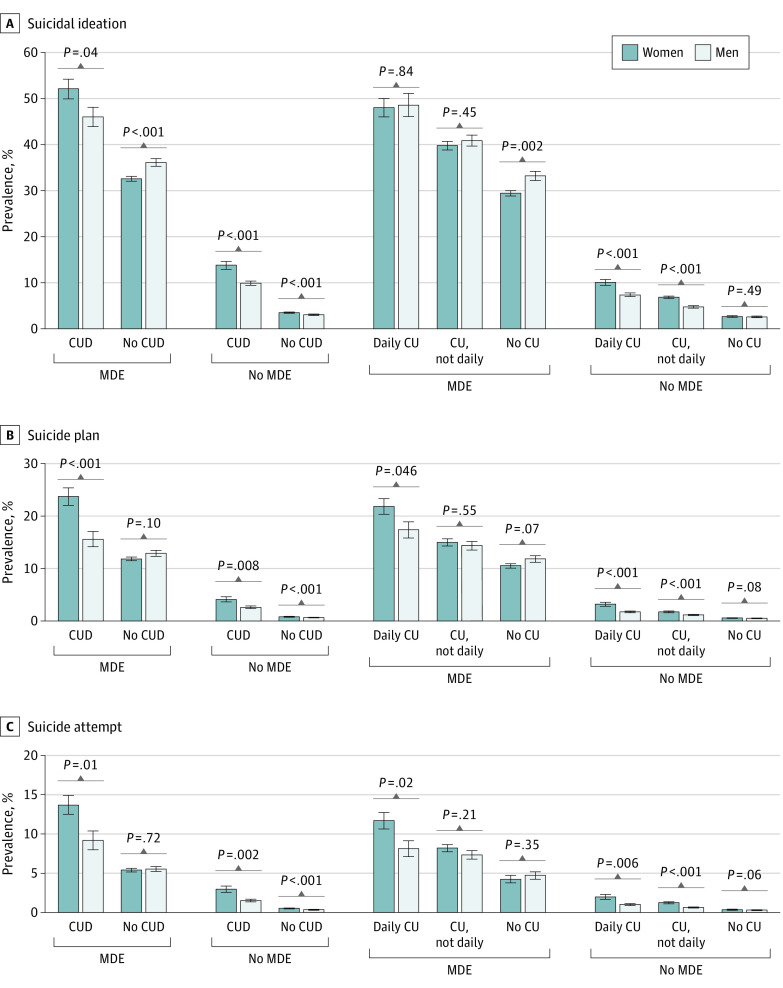
Adjusted Past-Year Prevalence of Suicidal Ideation, Suicide Plan, and Suicide Attempt by Depression, Cannabis Use (CU) and CU Disorder (CUD), and Sex Data are stratified by sex, major depressive episode (MDE), CU, and CUD. Daily CU indicates 300 or more days per year. Estimates were additionally adjusted for survey year, age, race/ethnicity, educational attainment/school enrollment, family income, employment status, marital status, health insurance status, nicotine dependence, alcohol use disorder, and cocaine use disorder. Error bars indicate 95% CI.

## Discussion

Using nationally representative data, we found that trends in suicidal ideation, plan, and attempt varied by the pattern of cannabis use (daily and nondaily cannabis use and CUD) among adults aged 18 to 34 years from 2008 to 2019, a time of marked increases in both cannabis use and suicidality. We found increases in suicidal ideation and plan and in daily cannabis use among every examined sociodemographic subgroup (except in daily cannabis use among current high school students) and increases in suicide attempt among most examined subgroups from 2008 to 2019.

Assessing both CUD and cannabis use status and their associations with suicidal ideation, plan, and attempt, we found that suicidality trends varied by sex, depression, and both CUD and cannabis use status. Our results suggest that CUD, daily cannabis use, and even nondaily cannabis use were associated with a higher prevalence of suicidal ideation, plan, and attempt more significantly in women than in men. Specifically, the adjusted prevalence of past-year suicidal ideation was higher among women with CUD regardless of MDE status and among women without MDE but with daily or nondaily cannabis use compared with their male counterparts. We found upward trends in suicidal ideation among women (rather than men) with MDE and CUD or daily and nondaily cannabis use. Compared with their male counterparts, the adjusted prevalence of suicide plan and attempt were higher among women with MDE and CUD or daily cannabis use and among women without MDE but with CUD or daily and nondaily cannabis use. Similarly, from 2008 to 2019, we found an upward trend in suicide plan among women (rather than men) with MDE and daily cannabis use and an upward trend in suicide attempt among women (rather than men) with MDE and CUD. By contrast, among individuals with neither MDE nor cannabis use, the adjusted prevalence of suicidal ideation, plan, and attempt were similar between men and women, and the adjusted prevalence of suicidal ideation was lower among women with MDE without CUD or cannabis use compared with their male counterparts.

Notably, from 2008 to 2019, the number of adults aged 18 to 34 years who died by suicide increased by 51.9% for women (from 1569 to 2384) and 44.9% for men (from 7266 to 10 529).^3^ Although adults with suicidality and adults who die by suicide can be interrelated yet distinct groups,^4,45^ our results are consistent with a possible role for cannabis use and CUD associated with the relatively higher percentage increase in deaths by suicide among women than men. Future research is needed to examine the associations highlighted in our study and assess the potential effect of cannabis use and CUD on suicide deaths among women compared with men, a phenomenon that is likely due to multiple factors.

Somewhat paradoxically, but consistent with earlier studies,^37,46^ we found that from 2008 to 2019, the prevalence of past-year CUD decreased among adults with daily cannabis use. This might reflect recent shifts toward greater acceptance of cannabis use, influencing perceptions of problematic consequences from cannabis consumption that are used as part of the criteria for CUD diagnosis,^38,47^ although further research is needed.

Our results, along with those from a recent study,^48^ suggest that adults with MDE may be particularly vulnerable to cannabis use as beliefs in its therapeutic potential become more widespread and products become more accessible. Moreover, even after adjusting for depression, CUD, cannabis use status, and other potential confounding factors, we found that from 2008 to 2019 among adults aged 18 to 34 years, the adjusted prevalence of suicidal ideation increased 1.4-fold; suicide plan, 1.6-fold; and suicide attempt, 1.4-fold. Furthermore, even for those with neither MDE nor cannabis use, we found upward trends in suicidal ideation and plan among both men and women and in suicide attempt among men. Our results indicate that depression and cannabis use are associated with suicidality but do not appear to be the only causes for the upward trends in suicide among young adults.

Death by suicide is a major public health problem in the US and a leading cause of mortality among US young adults. Among persons aged 15 to 54 years, approximately 60% of planned first suicide attempts occurred within the first year since the onset of suicidal ideation.^49^ People with a suicide plan constitute a psychiatric emergency, because suicide plan is associated with an imminent lethal attempt and a high risk of death.^10,50,51^ A suicide attempt history is the strongest clinical predictor of death by suicide.^4,30^ To improve the effectiveness of identifying and intervening with individuals who are at high risk of suicide, it is important to modify the specific risk factors associated with suicidality—including depression, cannabis use, and CUD—and to tailor interventions that are designed for women and other vulnerable populations. Previous studies^4,10,12,36,52,53,54^ have highlighted the importance of improving clinical insight and help-seeking and mental health treatment among individuals with MDE or suicidality. Because the prevalence of CUD increases with time since initiation of use among young adults,^55,56^ our results underscore an urgent need for prevention interventions designed specifically for young people before first cannabis exposure and highlight the importance of early screening for daily cannabis use and CUD as well as CUD treatment, especially among young women.

### Limitations

This study has several limitations. First, the prevalence of suicidal ideation, plan, and attempt may be underestimated because the NSDUH (1) did not account for people experiencing homelessness but not living in shelters, military personnel on active duty, and institutionalized populations and (2) is a self-reported survey subject to underreporting stigmatized behaviors (eg, suicidality) resulting from social desirability bias. Second, the endorsement of suicidal ideation and plan was based on single questions that could be interpreted differently by respondents. Third, the cross-sectional nature of NSDUH data precludes drawing causal inferences from reported associations. Research based on longitudinal data are needed to further examine and confirm our findings. Fourth, because the NSDUH does not collect data on anxiety and impulse-control disorders, we were unable to examine them; however, these disorders commonly co-occur with depression and CUD.^8^

## Conclusions

The results of this survey study indicate that CUD, daily cannabis use, and even nondaily cannabis use are associated with the risks of suicidal ideation, plan, and attempt in both young adult men and women, but significantly more so in women than men. Future research is needed to examine the increase in suicidality and to determine whether it is cannabis use or overlapping risk factors that increase risks for both.

## Notes

Supplement. **eTable 1.** Trends in the Prevalence of Past-Year Serious Thoughts of Suicide in US Adults Aged 18-34 Years (n = 279 886) **eTable 2.** Trends in Prevalence of Past-Year Suicide Plan Among US Adults Aged 18-34 Years (n = 279 861) **eTable 3.** Trends in Prevalence of Past-Year Suicide Attempt Among US Adults Aged 18-34 Years (n = 279 856) **eTable 4.** Trends in Prevalence of Past-Year Daily or Near-Daily Cannabis Use Among US Adults Aged 18-34 Years (n = 281 650) **eTable 5.** Trends in Prevalence of Past-Year Cannabis Use Disorder Among US Adults Aged 18-34 Years (n = 281 650) **eTable 6.** Correlates of Past-Year Suicidal Ideation, Suicide Plan, and Attempt Among US Adults Aged 18-34 Years
