# Supplementary material for: Associations of Suicidality Trends With Cannabis Use as a Function of Sex and Depression Status
Source: JAMA Netw Open. 2021 Jun 22;4(6):e2113025. doi: 10.1001/jamanetworkopen.2021.13025 (PMC8220498; doi:10.1001/jamanetworkopen.2021.13025)
Supplement: Supplement. — eTable 1. Trends in the Prevalence of Past-Year Serious Thoughts of Suicide in US Adults Aged 18-34 Years (n = 279 886) eTable 2. Trends in Prevalence of Past-Year Suicide Plan Among US Adults Aged 18-34 Years (n = 279 861) eTable 3. Trends in Prevalence of Past-Year Suicide Attempt Among US Adults Aged 18-34 Years (n = 279 856) eTable 4. Trends in Prevalence of Past-Year Daily or Near-Daily Cannabis Use Among US Adults Aged 18-34 Years (n = 281 650) eTable 5. Trends in Prevalence of Past-Year Cannabis Use Disorder Among US Adults Aged 18-34 Years (n = 281 650) eTable 6. Correlates of Past-Year Suicidal Ideation, Suicide Plan, and Attempt Among US Adults Aged 18-34 Years [file jamanetwopen-e2113025-s001.pdf]

Supplementary Online Content

Han B, Compton WM, Einstein EB, Volkow ND. Associations of suicidality trends with cannabis use as a function of sex and depression status. *JAMA Netw Open*. 2021;4(6):e2113025. doi:10.1001/jamanetworkopen.2021.13025

eTable 1. Trends in the Prevalence of Past-Year Serious Thoughts of Suicide in US Adults Aged 18-34 Years (n = 279 886)

eTable 2. Trends in Prevalence of Past-Year Suicide Plan Among US Adults Aged 18-34 Years (n = 279 861)

eTable 3. Trends in Prevalence of Past-Year Suicide Attempt Among US Adults Aged 18-34 Years (n = 279 856)

eTable 4. Trends in Prevalence of Past-Year Daily or Near-Daily Cannabis Use Among US Adults Aged 18-34 Years (n = 281 650)

eTable 5. Trends in Prevalence of Past-Year Cannabis Use Disorder Among US Adults Aged 18-34 Years (n = 281 650)

eTable 6. Correlates of Past-Year Suicidal Ideation, Suicide Plan, and Attempt Among US Adults Aged 18-34 Years

This supplementary material has been provided by the authors to give readers additional information about their work.

© 2021 Han B et al. *JAMA Network Open*.

eTable 1. Trends in the Prevalence of Past-Year Serious Thoughts of Suicide in US Adults Aged 18-34 Years (n = 279 886)

Weighted % (Standard Error)	2008-2009	2010-2011	2012-2013	2014-2015	2016-2017	2018-2019	*P* value for trend
**Overall**	5.3 (0.18)	5.3 (0.16)	5.8 (0.17)	6.2 (0.14)	7.3 (0.19)	8.7 (0.17)	<.001
**Age**							
18-23	6.8 (0.23)	7.3 (0.23)	8.0 (0.23)	8.6 (0.25)	10.2 (0.30)	12.4 (0.31)	<.001
24-29	4.7 (0.24)	4.7 (0.26)	4.6 (0.30)	5.5 (0.22)	6.4 (0.28)	8.2 (0.30)	<.001
30-34	4.1 (0.37)	3.3 (0.28)	4.3 (0.29)	4.3 (0.28)	4.8 (0.30)	5.3 (0.29)	<.001
**Sex**							
Men	4.7 (0.25)	4.7 (0.22)	5.3 (0.23)	5.7 (0.20)	6.8 (0.26)	7.6 (0.21)	<.001
Women	5.9 (0.23)	5.9 (0.22)	6.3 (0.23)	6.8 (0.21)	7.7 (0.25)	9.9 (0.26)	<.001
**Race/Ethnicity**							
Non-Hispanic white	5.8 (0.23)	5.6 (0.24)	6.2 (0.21)	6.9 (0.22)	8.1 (0.24)	9.7 (0.25)	<.001
Non-Hispanic Black	5.1 (0.39)	5.6 (0.36)	5.0 (0.39)	4.5 (0.31)	6.3 (0.39)	7.0 (0.39)	<.001
Hispanic	4.1 (0.32)	3.9 (0.29)	5.0 (0.43)	5.6 (0.37)	5.9 (0.36)	7.7 (0.43)	<.001
Non-Hispanic other	4.9 (0.46)	5.8 (0.50)	6.4 (0.66)	6.1 (0.48)	7.0 (0.47)	8.0 (0.51)	<.001
**Family Income**							
≤$20,000	6.5 (0.34)	6.8 (0.34)	7.4 (0.36)	8.0 (0.25)	9.3 (0.35)	11.3 (0.43)	<.001
$20000-$49,999	5.4 (0.25)	5.3 (0.26)	5.5 (0.28)	6.6 (0.31)	7.7 (0.29)	9.2 (0.29)	<.001
$50,000-$74,999	4.8 (0.41)	4.5 (0.35)	5.0 (0.36)	4.9 (0.36)	6.8 (0.45)	7.7 (0.41)	<.001
≥$75,000	4.5 (0.40)	4.1 (0.27)	5.0 (0.34)	5.2 (0.30)	5.6 (0.27)	7.4 (0.29)	<.001
**Employment Status**							
Full-time employed	4.1 (0.22)	3.9 (0.19)	4.3 (0.21)	5.0 (0.18)	6.0 (0.21)	7.4 (0.23)	<.001
Part-time employed	6.3 (0.28)	6.3 (0.28)	7.4 (0.38)	7.9 (0.33)	9.0 (0.41)	11.2 (0.50)	<.001
Unemployed	8.0 (0.69)	8.5 (0.58)	7.7 (0.56)	8.9 (0.63)	10.0 (0.65)	11.8 (0.75)	<.001
Other	6.7 (0.43)	6.4 (0.36)	7.2 (0.41)	7.2 (0.36)	8.2 (0.38)	9.2 (0.38)	<.001
**College/School Enrollment**							
Full-time college student	5.7 (0.32)	6.1 (0.33)	6.8 (0.39)	7.0 (0.35)	8.7 (0.45)	10.4 (0.38)	<.001
Part-time college student	6.3 (0.65)	5.9 (0.66)	6.7 (0.58)	7.2 (0.49)	9.0 (0.66)	11.5 (0.79)	<.001
College graduate, no enrollment	3.3 (0.35)	3.7 (0.33)	3.9 (0.41)	4.1 (0.29)	4.3 (0.29)	5.7 (0.35)	<.001
Some college education, no enrollment now	5.3 (0.50)	4.9 (0.48)	5.6 (0.54)	6.5 (0.34)	8.2 (0.48)	10.1 (0.44)	<.001
High school graduate, no enrollment now	5.8 (0.34)	5.5 (0.33)	5.7 (0.33)	6.6 (0.29)	7.3 (0.28)	8.9 (0.37)	<.001
Current high school students	9.2 (0.74)	8.1 (0.64)	8.6 (0.87)	11.4 (1.07)	12.0 (1.18)	10.2 (1.13)	.02
< high school education, no enrollment now	5.3 (0.46)	5.5 (0.54)	6.3 (0.49)	5.8 (0.44)	7.4 (0.57)	7.8 (0.51)	<.001
**PY Daily Cannabis Use & MDE**							
Daily cannabis use and MDE	47.6 (5.46)	49.0 (6.31)	49.1 (4.25)	46.9 (3.34)	43.9 (3.20)	52.6 (3.05)	.59
Daily cannabis use, but no MDE	7.7 (0.95)	6.6 (0.65)	8.1 (0.78)	7.1 (0.62)	9.6 (0.83)	9.2 (0.70)	.01
PY nondaily cannabis use; had MDE	39.2 (2.08)	35.6 (1.99)	35.5 (2.23)	41.4 (2.02)	42.6 (1.31)	43.5 (1.45)	<.001
PY nondaily cannabis use; no MDE	4.6 (0.33)	5.1 (0.39)	5.6 (0.41)	5.9 (0.33)	5.5 (0.35)	6.8 (0.30)	<.001
No PY cannabis use, but had MDE	27.7 (1.53)	28.3 (1.29)	27.8 (1.48)	29.9 (0.99)	34.1 (1.17)	35.0 (0.95)	<.001
No PY cannabis use, no MDE	2.3 (0.13)	2.3 (0.12)	2.5 (0.13)	2.6 (0.13)	2.9 (0.15)	3.3 (0.17)	<.001
**PY CUD & MDE**							
CUD and MDE	47.3 (3.86)	47.6 (5.03)	48.2 (4.49)	45.5 (3.05)	53.4 (3.81)	50.8 (3.07)	.30
CUD, but no MDE	9.5 (0.93)	9.3 (0.82)	10.5 (1.12)	10.7 (1.24)	12.3 (0.95)	14.0 (1.00)	<.001
No CUD, but had MDE	30.6 (1.29)	30.1 (1.10)	30.1 (1.16)	33.9 (1.01)	36.5 (1.00)	38.9 (0.89)	<.001
No CUD, no MDE	2.7 (0.13)	2.8 (0.12)	3.1 (0.12)	3.3 (0.11)	3.6 (0.16)	4.1 (0.14)	<.001

Data source: the 2008-2019 National Surveys on Drug Use and Health. PY=past-year; Daily=daily/near daily; MDE=major depressive episode; CUD=cannabis user disorder.

© 2021 Han B et al. *JAMA Network Open*.

eTable 2. Trends in Prevalence of Past-Year Suicide Plan Among US Adults Aged 18-34 Years (n = 279 861)

Weighted Percentage (Standard Error)	2008-2009	2010-2011	2012-2013	2014-2015	2016-2017	2018-2019	*P* value for trend
**Overall**	1.5 (0.08)	1.5 (0.09)	1.8 (0.10)	1.8 (0.07)	2.3 (0.08)	2.7 (0.08)	<.001
**Age**							
18-23	2.0 (0.12)	2.1 (0.13)	2.7 (0.13)	2.8 (0.13)	3.6 (0.16)	3.9 (0.15)	<.001
24-29	1.2 (0.12)	1.5 (0.14)	1.4 (0.16)	1.5 (0.11)	1.8 (0.12)	2.7 (0.16)	<.001
30-34	1.0 (0.15)	0.9 (0.12)	1.2 (0.11)	1.1 (0.20)	1.3 (0.14)	1.4 (0.16)	.02
**Sex**							
Men	1.3 (0.13)	1.3 (0.09)	1.6 (0.14)	1.6 (0.11)	1.9 (0.12)	2.2 (0.13)	<.001
Women	1.6 (0.09)	1.8 (0.13)	2.0 (0.14)	2.1 (0.10)	2.6 (0.12)	3.2 (0.14)	<.001
**Race/Ethnicity**							
Non-Hispanic white	1.5 (0.11)	1.6 (0.13)	2.0 (0.12)	1.9 (0.11)	2.5 (0.11)	2.9 (0.13)	<.001
Non-Hispanic Black	1.6 (0.18)	1.8 (0.19)	1.8 (0.30)	1.6 (0.25)	2.0 (0.16)	2.5 (0.23)	.003
Hispanic	1.2 (0.17)	1.1 (0.14)	1.4 (0.21)	1.7 (0.16)	1.8 (0.16)	2.4 (0.19)	<.001
Non-Hispanic other	1.3 (0.18)	1.5 (0.38)	1.5 (0.29)	2.0 (0.24)	2.2 (0.29)	2.7 (0.20)	<.001
**Family Income**							
≤$20,000	2.0 (0.18)	2.3 (0.22)	2.5 (0.21)	2.8 (0.15)	3.2 (0.23)	3.8 (0.22)	<.001
$20000-$49,999	1.5 (0.14)	1.4 (0.13)	1.7 (0.15)	1.9 (0.15)	2.4 (0.16)	3.0 (0.22)	<.001
$50,000-$74,999	1.1 (0.17)	1.5 (0.19)	1.2 (0.18)	1.2 (0.19)	1.9 (0.20)	2.3 (0.20)	<.001
≥$75,000	1.1 (0.14)	0.9 (0.11)	1.6 (0.18)	1.3 (0.18)	1.6 (0.15)	2.0 (0.11)	<.001
**Employment Status**							
Full-time employed	0.9 (0.08)	0.9 (0.08)	1.2 (0.11)	1.3 (0.10)	1.7 (0.10)	2.1 (0.11)	<.001
Part-time employed	2.0 (0.26)	1.5 (0.12)	2.7 (0.29)	2.2 (0.19)	2.8 (0.19)	3.3 (0.23)	<.001
Unemployed	2.4 (0.29)	2.9 (0.36)	2.6 (0.25)	3.1 (0.37)	3.5 (0.28)	4.3 (0.42)	<.001
Other	2.1 (0.20)	2.5 (0.25)	2.3 (0.25)	2.4 (0.21)	2.8 (0.25)	3.4 (0.26)	<.001
**College/School Enrollment**							
Full-time college student	1.7 (0.20)	1.6 (0.17)	2.2 (0.25)	1.9 (0.15)	2.7 (0.22)	2.8 (0.20)	<.001
Part-time college student	1.4 (0.23)	1.5 (0.30)	2.0 (0.32)	2.0 (0.32)	2.7 (0.39)	3.8 (0.39)	<.001
College graduate, no enrollment	0.6 (0.12)	0.8 (0.14)	0.8 (0.15)	1.0 (0.14)	0.8 (0.11)	1.2 (0.15)	.01
Some college education, no enrollment now	1.4 (0.19)	1.4 (0.25)	2.0 (0.35)	1.8 (0.19)	2.4 (0.22)	3.2 (0.27)	<.001
High school graduate, no enrollment now	1.6 (0.18)	1.7 (0.15)	1.8 (0.15)	2.1 (0.16)	2.8 (0.21)	3.4 (0.27)	<.001
Current high school students	3.4 (0.54)	2.8 (0.40)	3.4 (0.50)	4.6 (0.51)	4.7 (0.72)	4.5 (0.76)	.01
< high school education, no enrollment now	1.8 (0.20)	2.1 (0.29)	2.0 (0.24)	2.1 (0.31)	2.7 (0.30)	2.8 (0.34)	.004
**PY Daily Cannabis Use & MDE**							
Daily cannabis use and MDE	17.4 (3.66)	14.0 (2.71)	21.3 (3.04)	20.1 (2.79)	17.4 (1.69)	22.4 (2.20)	.10
Daily cannabis use, but no MDE	2.0 (0.46)	1.9 (0.38)	2.5 (0.57)	1.5 (0.26)	2.7 (0.41)	2.2 (0.34)	.41
PY nondaily cannabis use; had MDE	12.9 (1.24)	15.8 (1.42)	14.4 (1.38)	14.4 (1.27)	15.8 (1.03)	15.7 (1.10)	.06
PY nondaily cannabis use; no MDE	1.0 (0.14)	1.1 (0.15)	1.5 (0.18)	1.7 (0.22)	1.3 (0.14)	1.7 (0.17)	.001
No PY cannabis use, but had MDE	10.2 (0.80)	9.6 (0.86)	9.9 (1.02)	10.1 (0.77)	12.6 (0.87)	12.7 (0.85)	.004
No PY cannabis use, no MDE	0.4 (0.04)	0.5 (0.06)	0.6 (0.06)	0.5 (0.05)	0.7 (0.06)	0.7 (0.06)	<.001
**PY CUD & MDE**							
CUD and MDE	17.7 (2.60)	19.4 (3.65)	23.9 (3.30)	18.1 (2.01)	17.0 (1.90)	20.5 (2.68)	.94
CUD, but no MDE	2.5 (0.50)	2.6 (0.40)	2.5 (0.60)	3.2 (0.51)	3.7 (0.66)	4.0 (0.61)	.004
No CUD, but had MDE	10.8 (0.73)	10.3 (0.71)	11.0 (0.83)	11.8 (0.76)	13.9 (0.69)	14.2 (0.57)	<.001
No CUD, no MDE	0.5 (0.04)	0.6 (0.06)	0.8 (0.06)	0.8 (0.06)	0.9 (0.05)	0.9 (0.06)	<.001

Data source: the 2008-2019 National Surveys on Drug Use and Health. PY=past-year; Daily=daily/near daily; MDE=major depressive episode; CUD=cannabis user disorder.

© 2021 Han B et al. *JAMA Network Open*.

eTable 3. Trends in Prevalence of Past-Year Suicide Attempt Among US Adults Aged 18-34 Years (n = 279 856)

Weighted Percentage (Standard Error)	2008-2009	2010-2011	2012-2013	2014-2015	2016-2017	2018-2019	*P* value for trend
**Overall**	0.8 (0.06)	0.8 (0.06)	0.9 (0.07)	1.0 (0.06)	1.1 (0.06)	1.2 (0.06)	<.001
**Age**							
18-23	1.3 (0.09)	1.3 (0.09)	1.5 (0.11)	1.6 (0.12)	2.0 (0.13)	2.0 (0.13)	<.001
24-29	0.7 (0.09)	0.6 (0.11)	0.6 (0.11)	0.7 (0.08)	0.8 (0.07)	1.0 (0.09)	.001
30-34	0.5 (0.13)	0.5 (0.10)	0.6 (0.15)	0.5 (0.09)	0.4 (0.08)	0.6 (0.10)	.86
**Sex**							
Men	0.8 (0.09)	0.6 (0.07)	0.7 (0.08)	0.8 (0.07)	1.0 (0.08)	1.0 (0.08)	.004
Women	0.9 (0.07)	1.0 (0.10)	1.1 (0.13)	1.1 (0.08)	1.2 (0.08)	1.5 (0.09)	<.001
**Race/Ethnicity**							
Non-Hispanic white	0.8 (0.07)	0.8 (0.07)	0.8 (0.07)	1.0 (0.07)	1.2 (0.09)	1.1 (0.08)	<.001
Non-Hispanic Black	1.0 (0.15)	1.0 (0.15)	1.4 (0.29)	0.8 (0.16)	1.3 (0.15)	1.5 (0.16)	.02
Hispanic	0.8 (0.16)	0.7 (0.09)	0.9 (0.16)	1.0 (0.12)	1.0 (0.11)	1.2 (0.18)	.008
Non-Hispanic other	0.8 (0.15)	1.1 (0.33)	1.0 (0.23)	1.0 (0.13)	1.0 (0.15)	1.4 (0.17)	.140
**Family Income**							
≤$20,000	1.2 (0.14)	1.3 (0.16)	1.4 (0.18)	1.6 (0.12)	1.6 (0.14)	1.8 (0.12)	<.001
$20000-$49,999	1.0 (0.12)	0.8 (0.09)	0.9 (0.10)	1.0 (0.12)	1.4 (0.12)	1.3 (0.12)	<.001
$50,000-$74,999	0.5 (0.12)	0.6 (0.11)	0.7 (0.16)	0.6 (0.12)	0.8 (0.13)	0.9 (0.13)	.02
≥$75,000	0.6 (0.09)	0.4 (0.07)	0.6 (0.12)	0.6 (0.09)	0.7 (0.08)	0.9 (0.09)	<.001
**Employment Status**							
Full-time employed	0.5 (0.07)	0.4 (0.05)	0.5 (0.06)	0.6 (0.06)	0.8 (0.06)	0.9 (0.07)	<.001
Part-time employed	1.1 (0.19)	0.8 (0.10)	1.3 (0.21)	1.0 (0.14)	1.3 (0.14)	1.2 (0.12)	.23
Unemployed	1.6 (0.19)	1.6 (0.23)	1.5 (0.21)	2.0 (0.26)	2.3 (0.24)	2.9 (0.36)	<.001
Other	1.2 (0.16)	1.5 (0.16)	1.3 (0.18)	1.4 (0.17)	1.4 (0.16)	1.6 (0.16)	.14
**College/School Enrollment**							
Full-time college student	0.9 (0.16)	0.8 (0.11)	1.0 (0.18)	0.9 (0.13)	1.1 (0.15)	1.2 (0.12)	.070
Part-time college student	0.9 (0.17)	0.7 (0.14)	0.7 (0.17)	1.1 (0.21)	1.5 (0.31)	1.6 (0.31)	<.001
College graduate, no enrollment	0.1 (0.05)	0.3 (0.11)	0.3 (0.14)	0.5 (0.10)	0.2 (0.05)	0.3 (0.07)	.44
Some college education, no enrollment now	0.7 (0.13)	0.9 (0.17)	0.8 (0.24)	0.9 (0.14)	1.1 (0.15)	1.3 (0.16)	.004
High school graduate, no enrollment now	1.1 (0.14)	0.8 (0.10)	1.0 (0.10)	1.2 (0.13)	1.6 (0.13)	1.6 (0.14)	<.001
Current high school students	2.3 (0.34)	1.8 (0.36)	2.1 (0.39)	3.0 (0.50)	2.9 (0.50)	2.5 (0.61)	.16
< high school education, no enrollment now	1.3 (0.19)	1.4 (0.24)	1.7 (0.26)	1.1 (0.20)	1.6 (0.25)	2.0 (0.29)	.12
**PY Daily Cannabis Use & MDE**							
Daily cannabis use and MDE	11.8 (2.90)	8.3 (2.08)	12.9 (2.81)	9.3 (1.99)	8.7 (1.31)	9.6 (1.49)	.45
Daily cannabis use, but no MDE	1.6 (0.38)	1.4 (0.35)	0.9 (0.31)	0.8 (0.16)	1.7 (0.25)	1.2 (0.24)	.89
PY nondaily cannabis use; had MDE	9.8 (1.36)	6.6 (0.94)	7.8 (1.03)	8.2 (0.75)	8.1 (0.89)	7.1 (0.71)	.27
PY nondaily cannabis use; no MDE	0.6 (0.12)	0.8 (0.10)	1.1 (0.18)	0.9 (0.13)	0.7 (0.10)	0.9 (0.12)	.43
No PY cannabis use, but had MDE	4.4 (0.60)	4.5 (0.63)	3.9 (0.63)	4.4 (0.52)	4.6 (0.49)	4.5 (0.41)	.69
No PY cannabis use, no MDE	0.2 (0.04)	0.3 (0.05)	0.3 (0.05)	0.3 (0.04)	0.4 (0.05)	0.4 (0.05)	.002
**PY CUD & MDE**							
CUD and MDE	12.1 (2.85)	10.1 (1.84)	12.6 (2.21)	11.2 (1.84)	10.7 (1.73)	10.8 (1.70)	.73
CUD, but no MDE	1.8 (0.44)	2.0 (0.37)	1.6 (0.52)	1.7 (0.32)	2.4 (0.45)	2.1 (0.37)	.37
No CUD, but had MDE	5.8 (0.54)	4.9 (0.48)	5.0 (0.64)	5.5 (0.47)	5.7 (0.47)	5.5 (0.37)	.63
No CUD, no MDE	0.3 (0.03)	0.4 (0.04)	0.4 (0.04)	0.5 (0.04)	0.5 (0.05)	0.5 (0.05)	<.001

Data source: the 2008-2019 National Surveys on Drug Use and Health. PY=past-year; Daily=daily/near daily; MDE=major depressive episode; CUD=cannabis user disorder.

© 2021 Han B et al. *JAMA Network Open*.

eTable 4. Trends in Prevalence of Past-Year Daily or Near-Daily Cannabis Use Among US Adults Aged 18-34 Years (n = 281 650)

Weighted Percentage (Standard Error)	2008-2009	2010-2011	2012-2013	2014-2015	2016-2017	2018-2019	*P* value for trend
**Overall**	3.8 (0.14)	4.4 (0.13)	5.0 (0.17)	5.5 (0.15)	6.0 (0.13)	7.0 (0.16)	<.001
**Age**							
18-23	5.4 (0.17)	5.7 (0.16)	6.6 (0.23)	7.0 (0.27)	6.8 (0.22)	7.5 (0.23)	<.001
24-29	3.5 (0.24)	4.3 (0.24)	4.5 (0.28)	5.5 (0.26)	6.4 (0.21)	7.7 (0.27)	<.001
30-34	2.1 (0.24)	2.7 (0.33)	3.4 (0.37)	3.6 (0.24)	4.7 (0.24)	5.6 (0.35)	<.001
**Sex**							
Men	5.5 (0.24)	6.4 (0.24)	7.0 (0.28)	7.5 (0.26)	7.9 (0.22)	8.9 (0.28)	<.001
Women	2.2 (0.15)	2.3 (0.13)	3.0 (0.15)	3.5 (0.17)	4.2 (0.18)	5.0 (0.18)	<.001
**Race/Ethnicity**							
Non-Hispanic white	4.4 (0.19)	4.8 (0.18)	5.5 (0.20)	6.0 (0.21)	6.7 (0.21)	7.6 (0.26)	<.001
Non-Hispanic Black	4.5 (0.38)	4.9 (0.47)	6.3 (0.58)	6.7 (0.41)	7.1 (0.41)	8.7 (0.41)	<.001
Hispanic	2.3 (0.24)	3.2 (0.33)	3.5 (0.31)	4.1 (0.21)	4.7 (0.34)	5.3 (0.37)	<.001
Non-Hispanic other	1.9 (0.20)	2.7 (0.38)	3.1 (0.35)	3.4 (0.33)	4.1 (0.45)	5.1 (0.43)	<.001
**Family Income**							
≤$20,000	4.6 (0.27)	5.2 (0.30)	6.5 (0.40)	6.8 (0.31)	7.5 (0.32)	7.9 (0.39)	<.001
$20000-$49,999	4.4 (0.24)	5.1 (0.32)	5.1 (0.24)	6.1 (0.31)	6.6 (0.24)	7.9 (0.30)	<.001
$50,000-$74,999	3.2 (0.28)	3.7 (0.34)	4.4 (0.42)	4.6 (0.28)	5.9 (0.39)	7.5 (0.44)	<.001
≥$75,000	2.8 (0.25)	2.9 (0.21)	3.8 (0.28)	4.1 (0.23)	4.5 (0.18)	5.4 (0.25)	<.001
**Employment Status**							
Full-time employed	3.5 (0.20)	3.8 (0.19)	4.7 (0.22)	5.4 (0.23)	5.9 (0.17)	7.2 (0.23)	<.001
Part-time employed	4.3 (0.34)	4.9 (0.34)	5.5 (0.34)	5.6 (0.34)	6.5 (0.28)	6.7 (0.41)	<.001
Unemployed	6.2 (0.44)	7.4 (0.57)	7.6 (0.56)	8.2 (0.59)	9.2 (0.54)	11.3 (0.74)	<.001
Other	3.1 (0.25)	3.7 (0.27)	3.8 (0.29)	4.4 (0.33)	4.9 (0.32)	5.0 (0.28)	<.001
**College/School Enrollment**							
Full-time college student	3.8 (0.28)	3.9 (0.29)	4.5 (0.29)	4.6 (0.33)	4.5 (0.31)	4.8 (0.33)	.004
Part-time college student	3.8 (0.43)	5.0 (0.65)	4.1 (0.35)	5.4 (0.49)	6.1 (0.63)	6.2 (0.63)	<.001
College graduate, no enrollment	1.6 (0.27)	1.7 (0.23)	2.1 (0.24)	2.5 (0.25)	3.4 (0.31)	4.3 (0.25)	<.001
Some college education, no enrollment now	3.8 (0.42)	4.3 (0.42)	5.3 (0.48)	6.5 (0.41)	7.9 (0.39)	9.6 (0.48)	<.001
High school graduate, no enrollment now	4.9 (0.31)	5.7 (0.35)	6.6 (0.41)	7.2 (0.33)	8.0 (0.45)	9.1 (0.41)	<.001
Current high school students	4.6 (0.51)	4.0 (0.44)	4.5 (0.48)	5.0 (0.65)	4.1 (0.63)	4.7 (0.57)	.69
< high school education, no enrollment now	5.4 (0.33)	6.9 (0.47)	8.1 (0.57)	7.7 (0.51)	8.2 (0.63)	8.2 (0.58)	<.001
**Past-Year Cannabis Use Disorder**							
Yes	36.3 (1.36)	40.1 (1.66)	38.5 (1.87)	41.7 (1.60)	45.0 (1.53)	45.5 (1.54)	<.001
No	2.5 (0.11)	3.0 (0.11)	3.7 (0.16)	4.2 (0.13)	4.7 (0.12)	5.3 (0.14)	<.001
**Past-Year Major Depressive Episode**							
Yes	5.0 (0.59)	5.9 (0.72)	6.6 (0.55)	8.9 (0.64)	10.0 (0.62)	11.3 (0.59)	<.001
No	3.7 (0.15)	4.2 (0.14)	4.8 (0.18)	5.1 (0.16)	5.6 (0.14)	6.3 (0.17)	<.001
**Past-Year Serious thoughts of Suicide**							
Yes	8.7 (0.80)	9.2 (0.87)	10.9 (0.81)	11.2 (0.78)	12.8 (0.89)	14.2 (0.76)	<.001
No	3.5 (0.14)	4.1 (0.14)	4.6 (0.17)	5.1 (0.15)	5.5 (0.13)	6.3 (0.16)	<.001
**Past-Year Suicide Plan**							
Yes	9.8 (1.67)	9.3 (1.17)	12.7 (1.80)	12.4 (1.40)	13.8 (1.27)	16.2 (1.26)	<.001
No	3.8 (0.14)	4.3 (0.13)	4.8 (0.16)	5.3 (0.14)	5.9 (0.13)	6.7 (0.16)	<.001
**Past-Year Suicide Attempt**							
Yes	12.4 (1.98)	1.4 (1.88)	12.5 (2.36)	11.2 (1.65)	15.4 (1.72)	16.6 (1.91)	0.04
No	3.8 (0.14)	4.3 (0.13)	4.9 (0.17)	5.4 (0.15)	5.9 (0.13)	6.8 (0.16)	<.001

Data source: the 2008-2019 National Surveys on Drug Use and Health.

© 2021 Han B et al. *JAMA Network Open*.

eTable 5. Trends in Prevalence of Past-Year Cannabis Use Disorder Among US Adults Aged 18-34 Years (n = 281 650)

Weighted Percentage (Standard Error)	2008-2009	2010-2011	2012-2013	2014-2015	2016-2017	2018-2019	*P* value for trend
**Overall**	3.9 (0.13)	3.7 (0.12)	3.7 (0.13)	3.5 (0.11)	3.4 (0.13)	4.2 (0.15)	.84
**Age**							
18-23	6.3 (0.21)	6.2 (0.21)	5.9 (0.23)	5.4 (0.21)	5.5 (0.23)	6.4 (0.25)	.26
24-29	3.0 (0.22)	3.0 (0.22)	2.5 (0.19)	3.0 (0.21)	2.9 (0.20)	3.8 (0.26)	.03
30-34	1.7 (0.21)	1.5 (0.19)	2.1 (0.24)	1.6 (0.13)	1.4 (0.14)	2.0 (0.18)	.57
**Sex**							
Men	5.2 (0.20)	5.1 (0.19)	5.1 (0.22)	4.7 (0.21)	4.6 (0.21)	5.4 (0.24)	.72
Women	2.5 (0.13)	2.4 (0.13)	2.3 (0.13)	2.3 (0.09)	2.2 (0.12)	2.9 (0.15)	.29
**Race/Ethnicity**							
Non-Hispanic white	4.0 (0.17)	3.6 (0.16)	3.7 (0.18)	3.4 (0.14)	3.3 (0.16)	4.1 (0.19)	.93
Non-Hispanic Black	4.6 (0.40)	5.1 (0.40)	5.4 (0.40)	4.9 (0.28)	5.0 (0.32)	4.9 (0.41)	.84
Hispanic	3.6 (0.28)	3.6 (0.28)	3.0 (0.30)	3.1 (0.30)	3.0 (0.26)	3.9 (0.30)	.98
Non-Hispanic other	2.6 (0.28)	2.9 (0.38)	2.8 (0.37)	2.7 (0.29)	2.4 (0.24)	3.8 (0.36)	.09
**Family Income**							
≤$20,000	5.1 (0.31)	5.0 (0.30)	5.0 (0.26)	4.4 (0.25)	4.5 (0.25)	5.3 (0.36)	.63
$20000-$49,999	3.9 (0.19)	3.7 (0.22)	3.4 (0.18)	3.7 (0.20)	3.4 (0.17)	3.9 (0.20)	.99
$50,000-$74,999	2.9 (0.31)	2.8 (0.22)	2.8 (0.24)	2.9 (0.26)	2.9 (0.28)	3.9 (0.30)	.03
≥$75,000	3.5 (0.25)	3.1 (0.22)	3.5 (0.33)	2.8 (0.21)	2.8 (0.20)	3.8 (0.24)	.76
**Employment Status**							
Full-time employed	3.0 (0.14)	2.5 (0.15)	2.9 (0.17)	2.9 (0.16)	2.5 (0.13)	3.5 (0.16)	.03
Part-time employed	4.8 (0.31)	5.2 (0.39)	4.4 (0.24)	4.6 (0.30)	4.8 (0.30)	5.2 (0.33)	.69
Unemployed	7.1 (0.45)	7.1 (0.45)	6.9 (0.54)	5.6 (0.43)	7.3 (0.48)	8.0 (0.77)	.52
Other	3.9 (0.32)	3.7 (0.25)	3.5 (0.27)	3.0 (0.22)	2.9 (0.22)	3.5 (0.24)	.03
**College/School Enrollment**							
Full-time college student	5.2 (0.36)	4.6 (0.29)	4.8 (0.34)	4.2 (0.26)	4.2 (0.35)	5.2 (0.39)	.45
Part-time college student	4.0 (0.44)	4.3 (0.52)	3.8 (0.39)	4.5 (0.50)	4.3 (0.52)	4.0 (0.34)	.87
College graduate, no enrollment	1.6 (0.20)	1.5 (0.19)	1.4 (0.19)	1.7 (0.17)	1.8 (0.23)	2.8 (0.25)	<.001
Some college education, no enrollment now	2.4 (0.25)	2.8 (0.32)	3.1 (0.27)	3.6 (0.28)	3.4 (0.26)	4.6 (0.34)	<.001
High school graduate, no enrollment now	4.2 (0.26)	4.1 (0.24)	4.1 (0.26)	3.7 (0.24)	3.7 (0.27)	4.3 (0.29)	.59
Current high school students	7.2 (0.78)	6.6 (0.62)	7.7 (0.75)	5.1 (0.63)	5.1 (0.71)	4.8 (0.61)	<.001
< high school education, no enrollment now	5.7 (0.43)	5.2 (0.46)	4.6 (0.40)	3.9 (0.41)	3.9 (0.39)	4.4 (0.46)	.003
**Past-Year Daily/Near Daily Cannabis Use**							
Yes	36.6 (1.39)	34.3 (1.49)	28.3 (1.43)	26.5 (1.21)	25.2 (1.18)	27.0 (1.16)	<.001
No, but used cannabis in the past year	12.3 (0.43)	11.7 (0.47)	11.0 (0.53)	9.7 (0.38)	8.5 (0.37)	9.5 (0.41)	<.001
**Past-Year Major Depressive Episode**							
Yes	7.0 (0.53)	8.0 (0.60)	7.5 (0.62)	8.3 (0.60)	8.0 (0.54)	9.6 (0.50)	<.001
No	3.6 (0.13)	3.4 (0.12)	3.3 (0.13)	3.0 (0.12)	2.8 (0.11)	3.4 (0.14)	.01
**Past-Year Serious thoughts of Suicide**							
Yes	11.1 (0.90)	11.1 (0.81)	10.8 (0.88)	10.2 (0.58)	10.4 (0.76)	11.7 (0.68)	.78
No	3.5 (0.13)	3.3 (0.12)	3.2 (0.13)	3.0 (0.12)	2.8 (0.11)	3.4 (0.15)	.09
**Past-Year Suicide Plan**							
Yes	12.7 (1.63)	13.4 (1.70)	12.8 (1.66)	12.0 (1.16)	10.2 (0.97)	13.5 (1.25)	.75
No	3.7 (0.13)	3.6 (0.12)	3.5 (0.13)	3.3 (0.12)	3.2 (0.13)	3.9 (0.15)	.69
**Past-Year Suicide Attempt**							
Yes	15.3 (2.23)	15.1 (1.76)	14.4 (2.48)	13.0 (1.64)	13.1 (1.62)	15.6 (1.82)	.82
No	3.8 (0.13)	3.6 (0.12)	3.6 (0.13)	3.4 (0.12)	3.3 (0.13)	4.0 (0.15)	.97

Data source: the 2008-2019 National Surveys on Drug Use and Health.

© 2021 Han B et al. *JAMA Network Open*.

eTable 6. Correlates of Past-Year Suicidal Ideation, Suicide Plan, and Attempt Among US Adults Aged 18-34 Years

	Suicidal Ideation Adjusted Risk Ratio (95% CI) (n=279,886)	Suicide Plan Adjusted Risk Ratio (95% CI) (n=279,861)	Suicide Attempt Adjusted Risk Ratio (95% CI) (n=279,856)
**Year**			
2008-2009 +	1.0	1.0	1.0
2010-2011	1.0 (0.94-1.09)	1.1 (0.93-1.26)	1.0 (0.83-1.26)
2012-2013	**1.2 (1.13-1.33)**	**1.2 (1.08-1.45)**	1.1 (0.92-1.42)
2014-2015	**1.2 (1.13-1.29)**	**1.3 (1.12-1.46)**	**1.2 (1.02-1.47)**
2016-2017	**1.2 (1.09-1.25)**	**1.5 (1.33-1.71)**	**1.4 (1.17-1.67)**
2018-2019	**1.4 (1.26-1.53)**	**1.6 (1.45-1.85)**	**1.4 (1.17-1.67)**
**Age**			
18-23	**1.5 (1.41-1.58)**	**1.8 (1.56-2.07)**	**2.4 (1.98-2.91)**
24-29	**1.1 (1.07-1.19)**	**1.3 (1.14-1.43)**	**1.3 (1.08-1.58)**
30-34 +	1.0	1.0	1.0
**Sex**			
Men	1.0 (0.92-1.00)	**0.9 (0.85-0.98)**	**0.8 (0.73-0.90)**
Women +	1.0	1.0	1.0
**Race/Ethnicity**			
Non-Hispanic white +	1.0	1.0	1.0
Non-Hispanic Black	**0.9 (0.85-0.94)**	1.0 (0.92-1.15)	**1.3 (1.14-1.53)**
Hispanic	**0.9 (0.85-0.96)**	0.9 (0.87-1.03)	**1.2 (1.03-1.32)**
Non-Hispanic other	**1.1 (1.01-1.15)**	**1.1 (1.02-1.27)**	**1.4 (1.24-1.70)**
**College/School Enrollment**			
Full-time college student	1.0 (0.94-1.09)	**1.2 (1.003-1.43)**	**1.5 (1.03-2.04)**
Part-time college student	**1.2 (1.13-1.33)**	**1.5 (1.22-1.76)**	**1.9 (1.33-2.60)**
College graduate, no enrollment +	1.0	1.0	1.0
Some college education, no enrollment now	**1.2 (1.13-1.29)**	**1.5 (1.27-1.73)**	**1.9 (1.40-2.55)**
High school graduate, no enrollment now	**1.2 (1.09-1.25)**	**1.6 (1.37-1.84)**	**2.2 (1.62-2.87)**
Current high school students	**1.4 (1.26-1.53)**	**2.2 (1.81-2.62)**	**3.2 (2.28-4.43)**
< high school education, no enrollment now	**1.1 (1.03-1.23)**	**1.5 (1.30-1.82)**	**2.4 (1.79-3.28)**
**Family Income**			
<$20,000	**1.1 (1.02-1.15)**	**1.2 (1.05-1.27)**	1.1 (0.98-1.29)
$20000-$49,999	1.1 (1.00-1.12)	1.1 (0.95-1.17)	1.1 (0.96-1.27)
$50,000-$74,999	1.0 (0.96-1.09)	1.0 (0.88-1.09)	1.0 (0.81-1.14)
≥$75,000 +	1.0	1.0	1.0
**Employment Status**			
Full-time employed +	1.0	1.0	1.0
Part-time employed	**1.1 (1.06-1.17)**	**1.1 (1.02-1.27)**	1.1 (0.92-1.27)
Unemployed	**1.2 (1.11-1.28)**	**1.3 (1.14-1.44)**	**1.4 (1.21-1.61)**
Other	**1.2 (1.12-1.25)**	**1.3 (1.19-1.44)**	**1.3 (1.17-1.51)**
**Marital Status**			
Married +	1.0	1.0	1.0
Divorced or Separated	**1.3 (1.21-1.50)**	**1.6 (1.29-1.86)**	**1.6 (1.22-2.11)**
Never Married	**1.2 (1.15-1.30)**	**1.2 (1.05-1.36)**	1.0 (0.86-1.24)
Other	1.4 (0.95-1.91)	1.4 (0.84-2.47)	2.0 (1.00-4.12)
**Health Insurance**			
Private-only +	1.0	1.0	1.0
Uninsured	**1.1 (1.07-1.20)**	**1.2 (1.07-1.34)**	**1.4 (1.21-1.61)**
Medicaid	**1.1 (1.04-1.16)**	**1.2 (1.08-1.35)**	**1.5 (1.28-1.67)**
Other	**1.1 (1.07-1.21)**	**1.4 (1.21-1.54)**	**1.4 (1.19-1.66)**
**PY Major Depressive Episode (MDE)**			
Yes	**8.6 (8.22-8.94)**	**12.6 (11.70-13.62)**	**9.6 (8.63-10.67)**
No +	1.0	1.0	1.0
**PY Cannabis Use Disorder (CUD)**			
Yes	**1.4 (1.29-1.47)**	**1.3 (1.20-1.49)**	**1.4 (1.20-1.58)**
No +	1.0	1.0	1.0

© 2021 Han B et al. *JAMA Network Open*.

**PY Cannabis Use**			
Daily or near daily use	**1.5 (1.36-1.57)**	**1.5 (1.31-1.65)**	**1.5 (1.27-1.79)**
PY use, but no daily or nearly daily use	**1.3 (1.20-1.33)**	**1.2 (1.12-1.35)**	**1.4 (1.23-1.66)**
No PY use +	1.0	1.0	1.0
**Tobacco Use & Disorder**			
PM nicotine dependence (ND)	**1.2 (1.14-1.27)**	**1.5 (1.35-1.67)**	**1.9 (1.63-2.20)**
PY tobacco use, no PM ND	1.0 (0.96-1.04)	**1.1 (1.02-1.22)**	**1.2 (1.09-1.41)**
No PY tobacco use +	1.0	1.0	1.0
**Alcohol Use & Disorder**			
PY alcohol use disorder	**1.5 (1.45-1.65)**	**1.5 (1.33-1.65)**	**1.5 (1.28-1.78)**
PY alcohol use, no disorder	1.1 (1.00-1.10)	0.9 (0.86-1.03)	**0.9 (0.74-0.99)**
No PY alcohol use +	1.0	1.0	1.0
**Cocaine Use & Disorder**			
PY use or disorder	**1.2 (1.11-1.31)**	**1.2 (1.09-1.40)**	**1.4 (1.28-1.64)**
Lifetime use, no PY use	**1.1 (1.02-1.15)**	1.1 (0.95-1.16)	1.1 (0.93-1.24)
Never use+	1.0	1.0	1.0
**Interactions**			
**Sex x MDE x CUD**	*P*<0.001	*P*<0.001	*P*=0.05
In those with no MDE and no CUD: Men vs. Women	**0.8 (0.80-0.90)**	**0.7 (0.65-0.85)**	**0.7 (0.55-0.79)**
In women without CUD:MDE vs. No MDE	**7.7 (7.27-8.08)**	**10.9 (9.80-12.18)**	**8.2 (7.06-9.46)**
In women without MDE: CUD vs. No. CUD	**1.6 (1.36-1.84)**	**1.6 (1.26-2.13)**	**1.7 (1.20-2.27)**
**Sex x MDE x Cannabis Use**	*P*=0.003	*P*=0.001	*P*=0.01
In those with no MDE and no cannabis use: Men vs. Women	0.9 (0.87-1.02)	0.9 (0.73-1.06)	0.9 (0.72-1.14)
In women without cannabis use: MDE vs. No MDE	**8.8 (8.12-9.46)**	**13.4 (11.49-15.54)**	**9.7 (7.93-11.97)**
In women without MDE: Daily/Near Daily Use vs. No Use	**1.9 (1.59-2.15)**	**2.2 (1.70-2.87)**	**2.1 (1.39-3.13)**
PY Nondaily Cannabis Use vs. No Use	**1.6 (1.43-1.77)**	**1.8 (1.43-2.20)**	**2.1 (1.53-2.82)**
**Sex x MDE x Tobacco use & Disorder**	*P*=0.001	*P*<0.001	*P*=0.02
In those with no MDE and no tobacco use: Men vs. Women	1.0 (0.86-1.05)	**0.7 (0.56-0.87)**	**0.5 (0.35-0.66)**
In women without PY tobacco use: MDE vs. No MDE	**8.2 (7.64-8.89)**	**12.6 (10.93-14.59)**	**8.5 (6.70-10.70)**
In women without MDE: PM ND vs. No PY Tobacco Use	**1.4 (1.27-1.59)**	**1.7 (1.41-2.16)**	**1.8 (1.31-2.38)**
PY Tobacco Use, No PM ND vs. No PY Use	**1.1 (1.03-1.25)**	**1.3 (1.08-1.60)**	1.2 (0.89-1.60)
**MDE x Cocaine Use & Disorder**	*P*=0.01	*P*=0.003	*P*=0.01
In never cocaine users: MDE vs. No MDE	**9.0 (8.56-9.39)**	**13.5 (12.40-14.72)**	**10.5 (9.22-11.84)**
In those without MDE: Cocaine Use & Disorder vs. Never Use	**1.4 (1.23-1.57)**	**1.6 (1.32-1.95)**	**1.9 (1.50-2.34)**
Lifetime Use, No PY Use vs. Never Use	**1.1 (1.05-1.26)**	1.1 (0.96-1.34)	1.2 (0.90-1.49)
**MDE x Marital Status**			*P*=0.01
In married adults: MDE vs. No MDE			**14.2 (10.41-19.44)**
In those without MDE: Divorced/Separated vs. Married			**2.3 (1.36-3.72)**
Never Married vs. Married			1.3 (1.00-1.78)
Other vs. Married			**5.4 (2.48-11.60)**
**Age x Sex**	*P*=0.001		
For ages 29-34: Men vs. Women	1.1 (0.98-1.21)		
For women: Ages 18-23 vs. Ages 30-34	**1.6 (1.51-1.78)**		
Ages 24-29 vs Ages 30-34	**1.2 (1.08-1.27)**		
**Sex x Race/Ethnicity**	*P*=0.002		
In non-Hispanic whites: Men vs. Women	1.0 (0.96-1.05)		
In women: NH Black vs. NH white:	1.0 (0.91-1.06)		
Hispanic vs. NH white	0.9 (0.88-1.00)		
NH other vs. NH white	**1.1 (1.04-1.25)**		
**MDE x Alcohol Use & Disorder**	*P*<0.001		
In those without PY alcohol use: MDE Yes vs. No	**9.9 (9.02-10.94)**		
In those without MDE: Alcohol Use Disorder vs. no PY Use	**1.9 (1.71-2.15)**		
PY Alcohol Use but No Disorder vs. no PY Use	**1.1 (1.01-1.20)**		
**Sex x MDE & Employment Status**	*P*=0.01		
In those with full-time job, but no MDE: Men vs. Women	**0.7 (0.71-0.84)**		
In women with full-time job: MDE vs. No MDE	**7.8 (7.17-8.46)**		
In women without MDE: Part-Time Employed vs. Full-Time	**1.1 (1.04-1.23)**		
Unemployed vs. Full-Time	**1.2 (1.08-1.43)**		
Other vs. Full-Time	1.1 (0.00-1.22)		

Data source: the 2008-2019 National Surveys on Drug Use and Health. PY=past year; PM=past month; NH= non-Hispanic. Each **bolded** estimate is statistically significantly (*P*<0.05) different from the estimate of the reference group (with + sign within each cell).

© 2021 Han B et al. *JAMA Network Open*.
